# How the London Hospital Organised and Suffered

**Published:** 1917-01-27

**Authors:** 


### ON THE NIGHT OF THE EXPLOSION.

### HOW THE LONDON HOSPITAL ORGANISED AND SUFFERED.

*Nearly fifty continuous years of hospital experience have revealed to the writer most things that can and do happen to hospitals. The largest Metropolitan hospital, the London, Whitechapel, on the 19th instant was fated to receive treatment, the novelty of which has never before been experienced. The object of all hospitals is to admit wounded, injured, and sick persons to treatment as the best guarantee of their speedy restoration to health. Their privilege is to succour, treat, tenderly care for and recover the patients who seek their aid. In the case of a great accident, the first advice of the disaster and the need for special preparation at the hospital, usually come by telephone. At the London Hospital on the 19th instant the messenger outstripped the telephone service, and announced his presence by the smashing of 231 windows in the main building. Then the sky was illuminated for several minutes, to such a far-reaching extent that, it seemed to the beholder as if the lid had been taken off an enormous furnace, or that there was a great volcano in eruption. After the light of this monstrous flame went out, within three minutes, there was the sound of an intense explosion.*

*We felt that this unique treatment of a great hospital, which was first damaged structurally to a considerable extent, and then made the House of Refuge for the victims of the greatest explosion, in a war factory, yet experienced in England, has an historical interest which should be preserved by the publication in* THE HOSPITAL, *of a full record of what happened on the memorable night of January 19, and how the London Hospital prepared for and met a terrible emergency. Mr. E. W. Morris, the House Governor, has kindly sent us the following graphic and supremely interesting account of what happened. It demonstrates the splendid organising power which the nation possesses, through our voluntary hospital system, for its safety and protection. It is a matter for thankfulness, that the latest reports from the hospital are, that those who suffered injury and were admitted, with the possible exception of one or two old people, are making good progress towards recovery.*

